# Targeting Interleukin-10 Restores Graft Microvascular Supply and Airway Epithelium in Rejecting Allografts

**DOI:** 10.3390/ijms23031269

**Published:** 2022-01-23

**Authors:** Shadab Kazmi, Mohammad Afzal Khan, Talal Shamma, Abdullah Altuhami, Hala Abdalrahman Ahmed, Abdullah Mohammed Assiri, Dieter Clemens Broering

**Affiliations:** 1 Transplantation Research and Innovation Department, Organ Transplant Centre of Excellence, King Faisal Specialist Hospital and Research Centre, Riyadh 12713, Saudi Arabia; skazmi@kfshrc.edu.sa (S.K.); tshamma@kfshrc.edu.sa (T.S.); abaltuhami@kfshrc.edu.sa (A.A.); dbroering@kfshrc.edu.sa (D.C.B.); 2 Comparative Medicine Department, King Faisal Specialist Hospital and Research Centre, Riyadh 12713, Saudi Arabia; halbasheer@kfshrc.edu.sa (H.A.A.); assiri@kfshrc.edu.sa (A.M.A.); 3 College of Medicine, Alfaisal University, Riyadh 12713, Saudi Arabia

**Keywords:** interleukin-10, microvascular leakiness, airway epithelium

## Abstract

Interleukin-10 (IL-10) is a vital regulatory cytokine, which plays a constructive role in maintaining immune tolerance during an alloimmune inflammation. Our previous study highlighted that IL-10 mediated immunosuppression established the immune tolerance phase and thereby modulated both microvascular and epithelial integrity, which affected inflammation-associated graft malfunctioning and sub-epithelial fibrosis in rejecting allografts. Here, we further investigated the reparative effects of IL-10 on microvasculature and epithelium in a mouse model of airway transplantation. To investigate the IL-10 mediated microvascular and epithelial repair, we depleted and reconstituted IL-10, and monitored graft microvasculature, airway epithelium, and associated repair proteins. Our data demonstrated that both untreated control allografts and IL-10 (−) allografts showed a significant early (d6) increase in microvascular leakiness, drop-in tissue oxygenation, blood perfusion, and denuded airway epithelium, which is associated with loss of adhesion protein Fascin-1 and β-catenin on vascular endothelial cells at d10 post-transplantation. However, IL-10 (+) promotes early microvascular and airway epithelial repair, and a proportional increase in endothelial Fascin-1, and β-catenin at d10 post-transplantation. Moreover, airway epithelial cells also express a significantly higher expression of FOXJ1 and β-catenin in syngrafts and IL-10 (+) allografts as compared to IL-10 (−) and untreated controls at d10 post-transplantation. Collectively, these findings demonstrated that IL-10 mediated microvascular and epithelial changes are associated with the expression of FOXJ1, β-catenin, and Fascin-1 proteins on the airway epithelial and vascular endothelial cells, respectively. These findings establish a potential reparative modulation of IL-10 associated microvascular and epithelial repair, which could provide a vital therapeutic strategy to facilitate graft repair in clinical settings.

## 1. Introduction

Interleukin-10 is an anti-inflammatory cytokine secreted mainly by regulatory cells and plays a vital role in cell and tissue repair [1,2]. IL-10 has been reported to modulate regulatory T cells (Tregs) and conserve FOXP3 expression and associated suppressive function [3,4]. In addition, IL-10 influences wound healing, tissue repair, angiogenesis, and antifibrotic properties as reported in several preclinical studies including transplantation [2,5,6,7,8,9,10,11,12,13,14,15,16,17,18,19]. 

Loss of microvascular flow has been associated with the onset of tissue hypoxia, ischemia, and progression of sub-epithelial fibrosis, which is the hallmark of chronic rejection [20,21,22,23]. Moreover, preclinical and clinical lung transplantation studies also demonstrated that Tregs and their associated mediators modulate both airway epithelium and vascular endothelium of grafted tissue, which effectively modulates the expression of various adhesion proteins on the surface of both airway epithelium and microvessels within the graft [22,23,24,25,26,27,28,29,30,31]. Inflammation-associated airway epithelial, ciliary loss, and vascular endothelial injury have been reported in various preclinical and clinical transplantation studies [22,23,32,33,34]. FOXJ1 has been associated with ciliogenesis in airway epithelium and affected largely due to the severe hypoxic and ischemic state of the tissue [34]. β-Catenin plays a vital role in cell survival and proliferation; however, little is known regarding its role in endothelial cells. Moreover, several adhesion proteins, especially β-catenin and Fascin-1, have been reported to play a crucial part in vascular and epithelial repair [35]. Inflammation-associated immune modulation affects the extracellular expression of both β-catenin and Fascin-1 on epithelial and endothelial cells, respectively, which is crucial to tissue remodeling during allograft rejection. Previously, we demonstrated that therapeutic doses of IL-10 are sufficient to affect tissue microvasculature, associated inflammatory, and regulatory gene expression in a mouse model of airway transplantation, which establishes a vital therapeutic scope of IL-10 to rescue rejecting transplants for future translational studies. However, deposition of inflammatory mediators has been associated with microvascular and epithelial injuries, but clear molecular changes that initiate the microvascular and epithelial injuries are not yet investigated. Herein, we aimed to investigate the effects of IL-10 on graft microvascular and epithelial repair during allograft rejection. 

## 2. Results

### 2.1. IL-10 Reconstitution Preserve Functional Microvascular Supply

**Figure 1:**
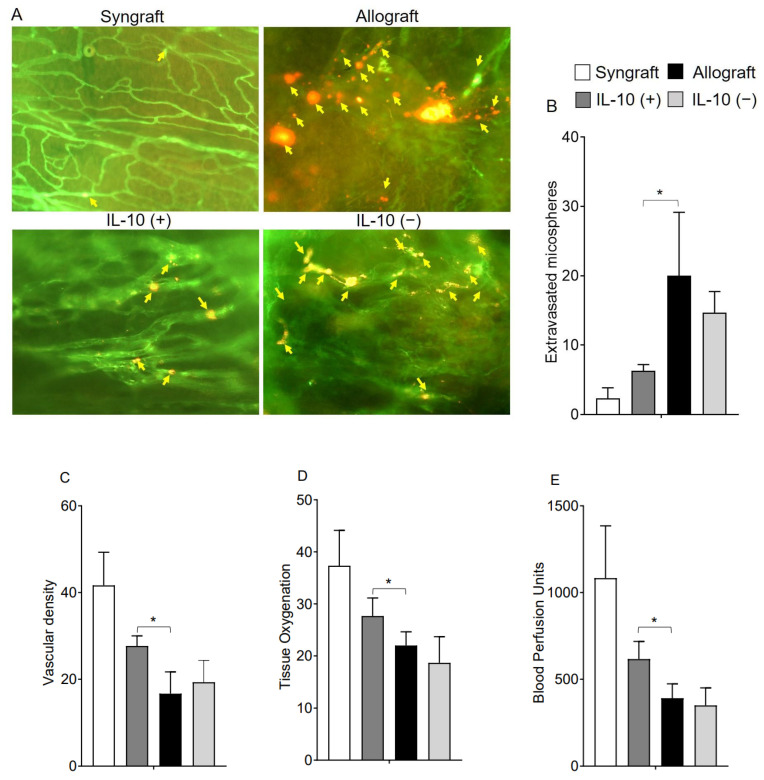
IL-10 is sufficient to preserve early microvascular leakiness. (**A**,**B**) microvascular leakiness, (**C**) microvascular density (**D**), tissue pO_2_ (mean ± SE, mmHg), and (**E**) blood perfusion units (mean ± SE, units) were plotted over different time points at d6 post-transplantation. Yellow arrows highlight extravasated microspheres in the graft. Data are presented as means with SE of 12 transplants/time point/experiment. * *p* < 0.05. Original magnification, ×20.

IL-10 is a vital reparative cytokine that plays a key role in donor microvasculature and, thereby, affect both hypoxic and ischemic state during rejection. While the effects of IL-10 blockade on immune suppression already emphasized the vital therapeutic significance in maintaining immune tolerance during transplant rejection [2,36], here, we further delineated the effects of IL-10 modulation on early microvascular leakiness, graft oxygenation, and microvascular blood flow during rejection. To test this, we performed microvascular leakiness and real-time measurements of tissue oxygenation (mmHg) and blood flow (Blood Perfusion Units) in syngrafts, untreated control allografts, as well as in IL-10 (−) and IL-10 (+) allografts at early d6 post-transplantation (Figure 1A–E). Our microvascular leakiness data demonstrated that both untreated control allografts and IL-10 (−) allografts showed a significant microvascular leakiness (*p* < 0.05) and consequently, showed a significantly hypoxic and ischemic phase (*p* < 0.05) as compared to corresponding syngrafts and IL-10 (+) allografts at d6 post-transplantation. These findings support the notion that IL-10 is directly/indirectly associated with microvascular reestablishment, which proportionally affects blood flow in the graft and oxygenation state during inflammation. (Figure 1A–E). 

### 2.2. IL-10 Reconstitution Suppress Goblet Cell Hyperplasia and Promote Ciliogenesis

**Figure 2:**
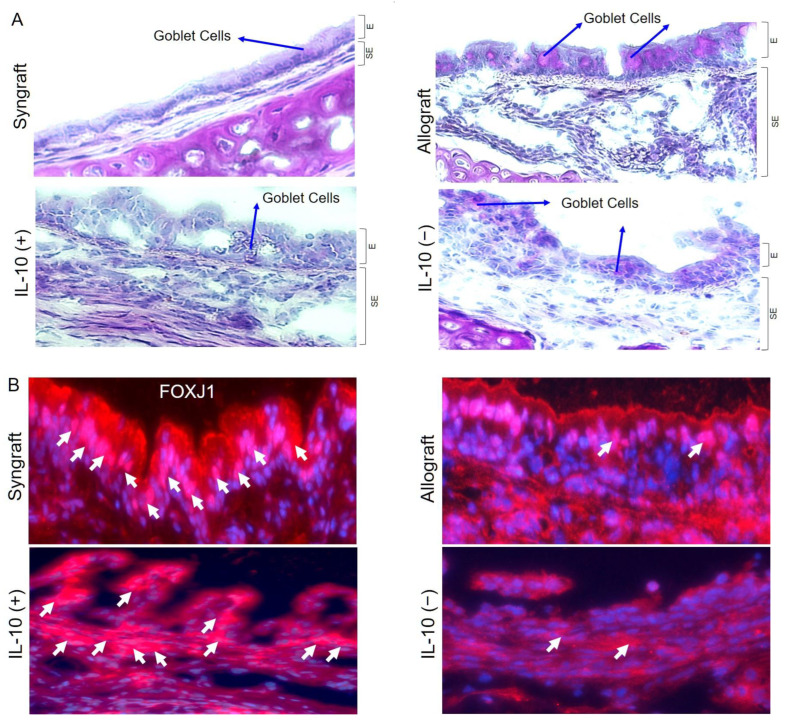

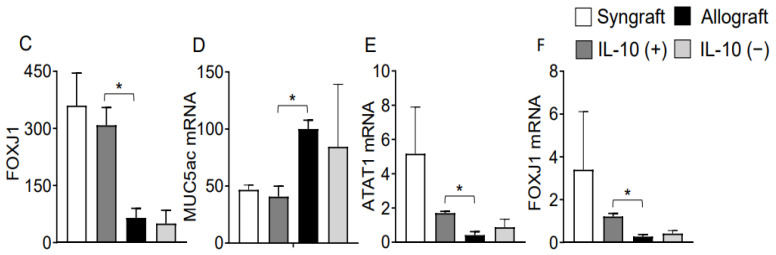
IL-10 is sufficient to suppress goblet cells hyperplasia and promote ciliogenesis. (**A**) PAS staining to highlight goblet cells in graft epithelium. (**B**,**C**) Immunofluorescence staining and semi-quantitative analysis of epithelial expression of FOXJ1 at d10 post-transplantation. (**D**–**F**) Quantitative RT-PCR analysis of grafts shows fold change in MUC5ac, ATAT1, and FOXJ1 mRNAs at d10 post-transplantation. Blue arrows highlight goblets cells, and white arrows highlight FOXJ1 expression in the epithelial region. Data are shown as means with SE 12 transplants/time point/experiment. * *p* < 0.05. Original magnification, ×40.

Microvasculature plays a crucial part in graft survival; therefore, these microvascular injuries impact the graft pathological structures during rejection. IL-10 takes an active part in tissue epithelium repair and wound healing, and thus, here, we investigated graft airway epithelium, specially goblet cells, after IL-10 mediated immunomodulation. To investigate the goblet cells hyperplasia, we performed Periodic acid–Schiff (PAS) staining of grafts and RT-PCR analysis in all experimental groups, which demonstrate a significant drop in epithelial goblet cells deposition post-IL-10 (+) compared to IL-10 (−) allografts at d10 post-transplantation (Figure 2A). Moreover, all corresponding tissue samples were immunostained for FOXJ1 expression (ciliated cells of airways) at d10 post-transplantation, and semi-quantitative analysis demonstrated a significant increase in FOXJ1 expression in syngrafts and IL-10 (+) allografts as compared to corresponding IL-10 (−) and untreated control allografts (Figure 2B).

**Figure 3:**
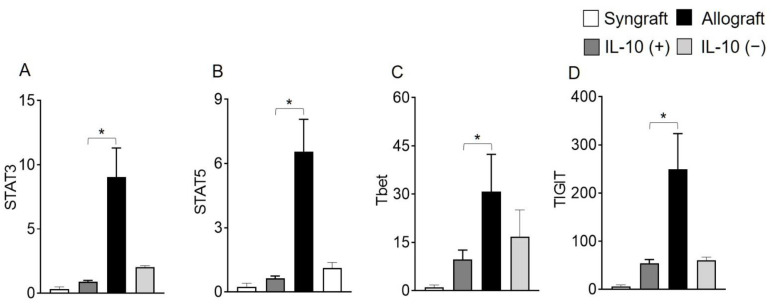

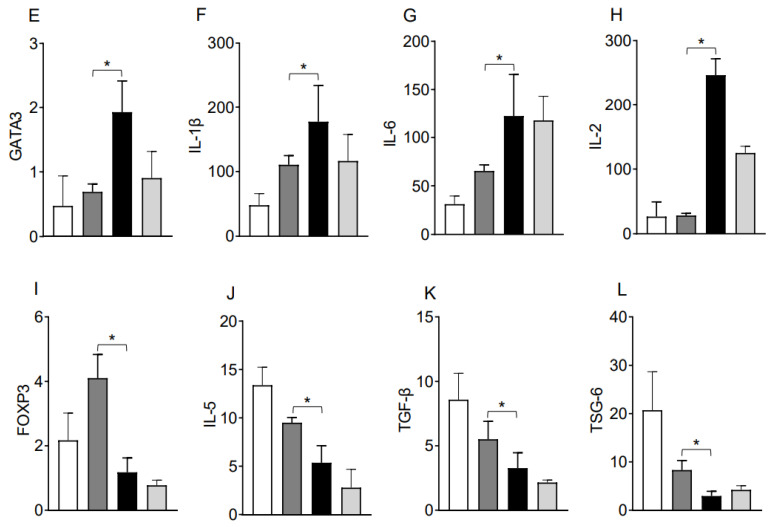
IL-10 is sufficient to promote the activation of regulatory genes. Quantitative RT-PCR analysis of grafts shows the fold change in mRNA of (**A**–**H**) inflammatory transcription factors and associated cytokines genes and (**I**–**L**) regulatory transcription factors and associated cytokines genes in control and IL-10-treated allografts at d10 post-transplantation. Data are shown as means with SE 12 transplants/time point/experiment. * *p* < 0.05.

Moreover, RT-PCR data of both syngrafts and IL-10 (+) allografts at d10 post-transplantation demonstrate an upregulation of regulatory phenotypes (FOXP3), associated regulatory mediators (IL-5, TGF-β, TSG-6) and airway epithelial repair mediators (ATAT1, and FOXJ1) as compared to untreated control allografts and IL-10 (−) allografts, which shows that IL-10 presence is sufficient to affect pathological changes. On the contrary, both untreated control allografts and IL-10 (−) allografts demonstrate an upregulation of major inflammatory phenotypes (STAT3, STAT5, Tbet, GATA3, and TIGIT), associated inflammatory mediators (IL-1β and IL-6), and airway epithelial injury mediator (MUC5ac) at the corresponding day post-transplantation (Figure 2C–F and Figure 3A–L). 

### 2.3. IL-10 Reconstitution Promote Epithelial and Endothelial Expression of Repair Proteins

**Figure 4:**
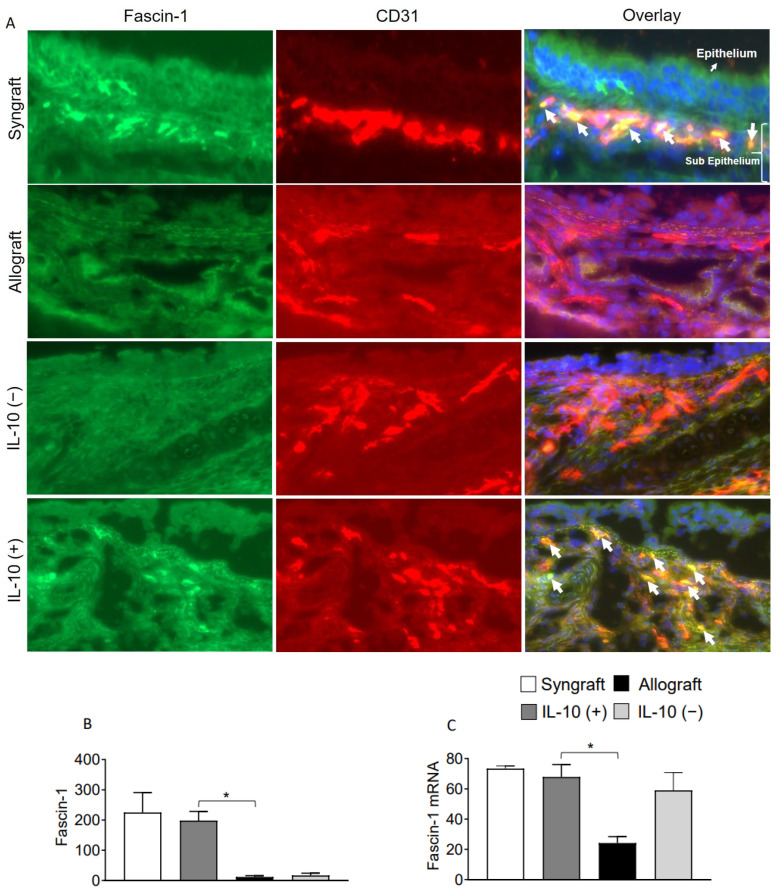
IL-10 is sufficient to promote vascular endothelial expression of Fascin-1. (**A**,**B**) Immunofluorescence staining and semi-quantitative analysis of graft expression of Fascin-1 on CD31^+^ vascular endothelial cells at d10 post-transplantation. (**C**) Quantitative RT-PCR analysis of grafts shows fold change in mRNA of Fascin-1 at d10 post-transplantation. White arrows highlight Fascin-1/CD31 coexpression in the subepithelial region. Data are presented as means with SE of 12 transplants/time point/experiment. * *p* < 0.05. Original magnification, ×40.

**Figure 5:**
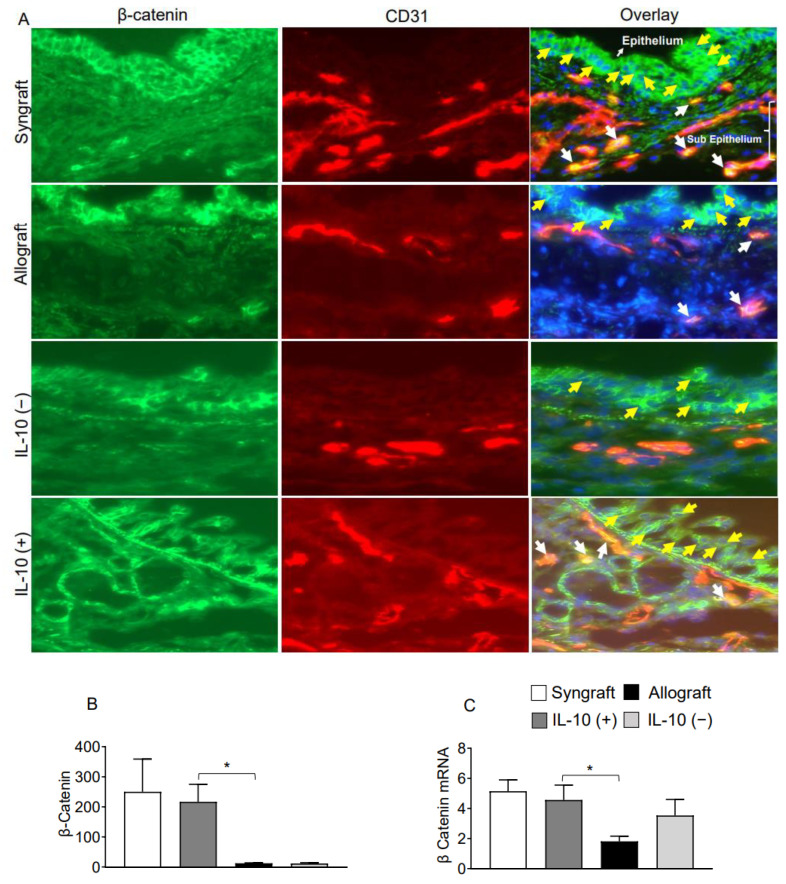
IL-10 is sufficient to promote vascular endothelial expression of β-catenin. (**A**,**B**) Immunofluorescence staining and semi-quantitative analysis of graft expression of β-catenin on CD31^+^ vascular endothelial cells at d10 post-transplantation (**C**) Quantitative RT-PCR analysis of grafts show fold change in mRNA of Fascin-1 at d10 post-transplantation. Yellow arrows highlight the β-catenin expression on the epithelial layer, and white arrows highlight β-catenin expression on CD31^+^ vascular endothelial cells. Yellow arrows highlight β-catenin expression in the epithelial, and white arrows highlight β-catenin/CD31 coexpression in subepithelial region. Data are presented as means with SE of 12 transplants/time point/experiment. * *p* < 0.05. Original magnification, ×40.

Interleukin-10 is generally known as an anti-inflammatory cytokine, but it also mediates a variety of functions at the vascular and endothelial structures. To validate this, we immunostained Fascin-1, β-catenin on CD31^+^ vascular endothelial cells. Immunofluorescence analysis demonstrates that IL-10 (+) allografts showed an upregulation of endothelial expression of Fascin-1 and β-catenin as compared to IL-10 (−) and untreated control allografts at d10 post-transplantation. Besides vascular endothelium, IL-10 (+) allografts also demonstrate an epithelial upregulation of β-catenin as compared to IL-10 (−) and untreated control allografts at d10 post-transplantation. To further confirm the graft expression of Fascin-1 and β-catenin, we tested all harvested transplants through RTPCR analysis, which demonstrated a significant overexpression of both Fascin-1 and β-catenin mRNA in syngrafts and IL-10 (+) allografts as compared to untreated allografts and IL-10 (−) allografts at d10 post-transplantation. These findings support the fact that enhanced regulatory activity in IL-10 (+) samples is pathologically correlated with both epithelial and endothelial repair, which allows graft functioning, see Figure 4A–C and Figure 5A–C.

## 3. Discussion

IL-10 is a vital immunoregulatory and wound-healing cytokine, which favors immune tolerance, and prolongs graft survival [2,10,11,13,37,38,39,40,41,42,43,44,45]. The anti-inflammatory, reparative, anti-fibrotic, and associated pathological effects of IL-10 on graft epithelial and microvascular structures have been reported in various preclinical settings [2,5,16,46]. Moreover, the immunomodulatory activity of IL-10 has been tested in various clinical studies [47,48,49]. Microvascular and epithelial injuries have been associated with lung transplant dysfunctions, which are crucial to the progression of chronic rejection [20,21,22,28,50,51,52,53,54,55,56,57,58,59,60,61]. The pathological studies of obstructive bronchitis patients further highlight that the epithelial and microvascular injuries were aggravated due to various cell and molecular mediators in affected distal airways, which obstructed the epithelial restoration and, thereby, augmented atypical tissue repair and fibro-proliferation [53,60,62,63]. IL-10 plays a constructive role in inflammation-associated repair post-transplantation, but the mechanisms through which IL-10 facilitates tissue repair, specifically epithelial and endothelial, is still not yet fully investigated. Here, we hypothesized that IL-10 (+) is sufficient to augment the expression of various wound healing and repair proteins on graft epithelial and endothelium, which supports both epithelial and microvascular repair. Therefore, the purpose of this study was to further investigate the effects of IL-10 on microvascular and epithelial repair post-transplantation. 

Overall, our results demonstrated that the occurrence of microvascular leakiness and associated hypoxia and ischemia at d6 post-transplantation directly correlated with epithelial and vascular loss as demonstrated by the overexpression of inflammatory cell phenotypes (STAT3, STAT5, GATA3, Tbet, and TIGIT) and associated inflammatory mediators (IL-6, IL-1 β, IL-2, and MUC5ac) at d10 post-transplantation. However, IL-10 (+) treatment augments the overexpression of regulatory cell phenotypes (FOXP3^+^), associated repair mediators (IL-5, TGF-β, TSG-6, FOXJ1, and ATAT1), and expression of Fascin-1 and β-catenin on vascular endothelial and airway epithelial cells, which paradoxically supports microvasculature and airway epithelial repair. These findings confirmed the notion that IL-10 is a vital reparative mediator to facilitate graft repair post-transplantation. Of note, IL-10 (+) treated allografts also demonstrated an upregulation of FOXJ1 expression, which has been associated with differentiation of airway epithelial cells, ciliogenesis, and phase of oxygenation [34,64]. 

Microvascular injuries of rejecting lung airways have been contributed to long-term organ health, and an effective immune response post-transplantation operates through both effector and regulatory phenotypes, which involves inflammatory and regulatory mediators, respectively [20,52,65,66,67,68]. Cytokines including IL-2, IL-1β, and IL-6 are potent inflammatory mediators, and play a decisive role in immune-mediated tissue destruction of the graft through the influx of mononuclear cells [69,70]. A discrete regulatory role of FOXP3^+^ expression and associated mediators (IL-5, TGF-β, and TSG-6) has been well demonstrated in maintaining the phase of immune tolerance and repair [2,71,72]. As reported earlier, pro-inflammatory cytokines, especially IL-6, have been shown to inhibit β-catenin expression [73,74], and β-catenin progressively regulates the activation of mTOR to augment dendritic cell-associated production of IL-10 [75]. Moreover, Fascin-1, an actin-bundling cytoskeletal regulatory protein, and β-catenin, a regulator of barrier integrity, is a key constituent of the adherens junctional complex, which could play a decisive role in maintaining microvascular and epithelial structures post-transplantation. Fascin-1 is mostly expressed in neurons, fibroblasts, endothelial, smooth muscle, dendritic, and mesenchymal cells [76]. Moreover, Fascin-1 plays a constructive role in cell development, migration, invasion, adhesion, and angiogenesis to facilitate tissue repair and wound healing [77]. Fascin-1 also binds β-catenin and colocalizes with it at the leading edges and borders of epithelial and endothelial cells [78,79]. In a mouse hind limb ischemia model, β-catenin significantly increased recovery of blood perfusion, capillary density along with enhanced VEGF expression, and the number of proliferating ECs and myocytes [80]. β-Catenin is an integral part of the alveolar epithelial cells (AEC) and takes a crucial part in lung repair [35,81,82]. β-Catenin is a key component to the patterning of the alveolar epithelium in development further revealed a prominent role for β-catenin in type II AEC differentiation, branching morphogenesis, alveolarization, lung repair, and protection from fibrosis [35,81,82,83,84]. β-Catenin has been reported to induce the expression of vascular endothelial growth factor (VEGF) and supports endothelial cells proliferation, survival, and angiogenesis [85,86,87,88,89,90]. Altogether, we demonstrated a crucial role of therapeutic IL-10 on FOXJ1, Fascin-1, and β-catenin in epithelial and endothelial repair, which paradoxically modulates graft health. These mediators affect graft microvascular and epithelial structures during the progression of chronic rejection, and therefore, maintenance of microvasculature is of crucial importance in solid organ transplantation.

Together, these results demonstrate that IL-10 upregulates regulatory phenotypes, which favors immune tolerance and repair, and thus, preserves the graft microvascular and epithelial structures. Our findings provide a proof-of-concept that IL-10 supports the revival of microvascular endothelium and airway epithelium through the surface expression of FOXJ1, Fascin-1, and β-catenin proteins and, thereby, preserve microvascular supply, tissue oxygenation, and airway epithelium in allografts. In conclusion, this therapeutic approach supports the idea that IL-10 is a wound healing mediator, and therefore, a combination therapy of IL-10 with other established therapeutic regimens would be an ideal option to control the inflammation associated graft injuries and contain the progression of chronic rejection. 

## 4. Materials and Methods

### 4.1. Donor and Recipients Mice Strains

Mice strains used in this transplantation study were originally sourced from the Jackson Laboratory (JAX, Bar Harbor, ME, USA), and maintained as an original colony in an animal research facility at King Faisal Specialist Hospital and Research Centre (KFSH&RC), Riyadh, Saudi Arabia. The KFSH&RC Animal Care and Use Committee (ACUC) approved the experimental protocol adopted in this study (RAC No. 2190024). In brief, C57BL/6J (B6.H-2b) mice strains were used as transplant donors for syngrafts and as recipients of all other allografts, while BALB/CJ (H-2d) strains were used as allogeneic transplant donors for C57BL/6J in all transplants. (Table 1).

### 4.2. Experimental Planning

This is a follow-up study of our previous published work, which showed that therapeutic intervention of IL-10 restored microvascular supply, and therefore, improved graft oxygenation and blood flow as compared to untreated control allografts at d10 post-transplantation [2]. These findings prompted us to further investigate ongoing microvascular injuries, and what molecular perturbations are involved in this phase of vascular injury. Therefore, in this study, we selected early d6 post-transplantation to investigate an ongoing microvascular injury in a well-established mouse model of orthotopic tracheal transplantation (OTT) [22,23,29,91]. We examined all IL-10-treated and control grafts for ongoing microvascular leakiness in functional microvasculature at d6 post-transplantation. Moreover, all IL-10-treated and control grafts were also examined using immunofluorescence imaging and gene transcripts at d10 post-transplantation to further correlate the expression of various targeted inflammatory, regulatory, and repair-associated markers [22,23,28,29,91,92]. The selection criteria of the individual day are based on the occurrence of specific molecular/pathological symptoms during transplantation, and as reported in earlier studies in this model, d6 is the point where tissue oxygenation/microvascular blood flow peaks, while d10 is the point of acute rejection with maximum lymphocytes infiltration, low tissue oxygenation, and no blood flow between donor-recipients [22,23,71,91]. Therefore, we selected only d6 and d10 in this follow-up study to investigate vascular and epithelial repair in IL-10 (+) allografts post-transplantation. 

### 4.3. Airway Surgical Procedure

Tracheal transplantation of allografts (BALB/CJ→C57BL/6J) and syngrafts (C57BL/6J →C57BL/6J) were performed under sterile conditions as described in the detailed procedure [91,93]. The recipient mouse was anesthetized (Ketamine 100 mg/kg, Xylazine 20 mg/kg), and the laryngotracheal area was prepared to connect donor trachea (6–8 rings) with 10-0 non-absorbable sterile suture (AROSurgical, Newport Beach, CA, USA) and, finally, the overlying skin was stitched with 5-0 non-absorbable sterile suture (AROSurgical, Newport Beach, CA, USA). As a part of post-operative care, all transplants received Carprofen (dose 5 mg/kg × SC) and Zolecin (dose 100 mg/kg × SC) medications and were monitored next 24 h for any respiratory distress.

### 4.4. IL-10 Depletion (−) and Reconstitution (+)

To achieve systemic IL-10 depletion, transplants were intraperitoneally injected with 250 μg/day of anti-mouse IL-10 (Bio X Cell, Lebanon, NH, USA) for at least 2 weeks after the day of transplantation [2,94]. However, to achieve systemic IL-10 reconstitution, transplants were intravenously injected with 4 μg of recombinant IL-10 (Pepro Teck, London, UK) in 100 μL of sterile PBS or with vehicle alone on days 3, 5, 7, and 9 post-transplantations [95]. An ELISA was run to confirm the serum levels of IL-10 post depletion and reconstitution experiments to validate the systemic IL-10 modulation.

### 4.5. Graft Microvascular Leakiness

To test the microvascular leakiness, d6 grafts were examined using intravenous injection of FITC-lectin and R50 Fluoro-Max red fluorescent microspheres (Thermo Fisher Scientific, Waltham, MA, USA) cocktail [22,23,28,96], and after 5 min in circulation, vasculature was washed with 1% PFA (paraformaldehyde), and grafts were harvested and incubated in 1% PFA at 4 °C for 10 min. Next, the grafts were mounted and examined through fluorescence microscopy (EVOS imaging system, Life Technologies, Paisley, UK) [22,23].

### 4.6. Graft Blood Flow, Oxygenation Analysis

Oxygen content (tpO2 mmHg) and blood flow (Blood perfusion Units; BPUs) in rejecting allografts were measured in real-time by OxyLite/Oxyflow combined sensors (model NX-BF/OF/E, Oxford Optronix, Milton, UK) as originally described [22,23,72,91]. In brief, transplanted mice were anesthetized and the airway graft was surgically exposed, and finally, the sensor was inserted through a 27G needle to touch the airway epithelium for oxygen and blood flow content [91,97,98]. Next, graft oxygenation and blood flow state were recorded at d6 post-transplantation. 

### 4.7. Immunofluorescence Analysis

Next, the grafts were harvested at d10 post-transplantation to further evaluate the extent of graft expression of FOXJ1, β-catenin, CD31, and Fascin-1 in IL-10-treated samples. Briefly, transplants were harvested and frozen in Tissue-Tek O.C.T. medium (Sakura Finetek, Tokyo, Japan) [71,72,98]. Next, frozen samples were sliced into 5 μm thin sections using a cryostat (Cryo 3, Sakura), and mounted on super frost/plus slides (Thermo Fisher Scientific, Waltham, MA, USA) for Immunofluorescence staining. In brief, slides were processed in methanol/acetone (1:1) and incubated with 10% donkey serum for 30 min and then incubated for 1 h with either rat-anti mouse FOXJ1 (Abcam, Waltham, MA, USA), rat anti-mouse β-catenin (Cell signaling Technology, Danvers, MA, USA), rat anti-mouse CD31, and rabbit anti-mouse Fascin-1 (Abcam, Waltham, MA, USA) primary antibodies. The slides were then washed with PBS, and sections were further incubated for 1 h with Alexa Fluor 488 labeled donkey anti-rabbit (Jackson ImmunoResearch, West Grove, PA, USA), Alexa Fluor 488 labeled donkey anti-rat (Jackson ImmunoResearch, West Grove, PA, USA) Cy3 labeled donkey anti-rat (Jackson ImmunoResearch, West Grove, PA, USA) secondary antibodies. After incubation, sections were washed and mounted in Vectashield mounting medium (Vector Laboratories, Burlingame, CA, USA). Immunofluorescence image analysis was performed with the EVOS FL auto cell imaging system (Life Technologies, Paisley, UK), and the percentage of co-localization was quantified through the mean integrated fluorescent intensity of Alexa 488 to detect either β-catenin or Fascin-1; furthermore, Cy3 was used to detect FOXJ1 and CD31 expression, while coexpression of selected proteins was quantified using ImageJ software [22,23,28]. 

### 4.8. PCR Analysis

RT-PCR analysis of pro-inflammatory, regulatory, epithelial, and vascular repair-associated genes was performed with some modifications [99]. Briefly, Total RNA from tracheal grafts was extracted using RNeasy mini kit 50 (Qiagen Sciences, Germantown, MD, USA) and quantified using a NanoDrop 1000 spectrophotometer (NanoDrop Technologies, Wilmington, DE, USA). cDNA from each isolated RNA was synthesized with a high-capacity cDNA reverse transcription kit (Applied Biosystems, Waltham, MA, USA) and real time-PCR was performed using gene-specific primers on an AB 7500 Fast Real-Time PCR system in triplicates (Applied Biosystems, Waltham, MA, USA) using Power SYBR Green (Applied Biosystems, Waltham, MA, USA). Data were analyzed with integrated software, and expression levels were analyzed with the 2^−∆∆Ct^ method after normalization to the housekeeping genes 18 s ribosomal RNA. We selected genes with expected regulatory, epithelium injury/repair, and inflammatory effects. A complete list of individual primers used in the present study is shown in Table 2.

### 4.9. Statistical Analysis

GraphPad™ Prizm software was used for statistical analysis to analyze different transplants over time. Differences between various groups at one-time points were compared by one-way ANOVA and a *p*-value < 0.05 was considered as significant.

## Figures and Tables

**Table 1:** Experimental groups.

Donor	Recipient	Treatment Plan	Monitoring of Transplants (Days)
C57BL/6	C57BL/6	Vehicle-Treated Syngraft Control	6,10
BALB/c	C57BL/6	Vehicle-Treated Allograft Control	6,10
BALB/c	C57BL/6	IL-10 Depletion	6,10
BALB/c	C57BL/6	IL-10 Reconstitution	6,10

**Table 2:** Details of primers used in RT PCR analysis.

Gene	Forward Primer	Reverse Primer
*STAT3*	GTCTGTAGAGCCATACACCAAG	GGTAGAGGTAGACAAGTGGAGA
*STAT5*	CGCTTCAGTGACTCGGAAAT	CAGGGACCGAATGGAGAAATC
*GATA3*	CTCGGCCATTCGTACATGGAA	CTCGGCCATTCGTACATGGAA
*Tbet*	AGCAAGGACGGCGAATGTT	GGGTGGACATATAAGCGGTTC
*TIGIT*	GCTGACCCACAGGAATACTTTA	GAGAGACATAGGGAGAGGGATAG
*FOXP3*	GTGGTTAGGAGACATCCATCAG	CTTTGAGCAACCTGGAGAAGA
*IL-2*	GCGGCATGTTCTGGATTTG	TGTGTTGTCAGAGCCCTTTAG
*IL-1β*	GGTGTGTGACGTTCCCATTA	ATTGAGGTGGAGAGCTTTCAG
*IL-5*	CTCTGTTGACAAGCAATGAGACG	TCTTCAGTATGTCTAGCCCCTG
*IL-6*	GTCTGTAGCTCATTCTGCTCTG	GAAGGCAACTGGATGGAAGT
*TGF- β*	CTGAACCAAGGAGACGGAATAC	GGGCTGATCCCGTTGATTT
*TSG-6*	GCTACAACCCACATGCAAAG	GACCTGGTTGTCATCGTACTC
*Fascin-1*	GGAACTCTGGCACCTTTCTT	CCAGTTACAAGCTCAGGGTAAG
*β -catenin*	GACACCTCCCAAGTCCTTTATG	CTGAGCCCTAGTCATTGCATAC
*ATAT1*	GGTCACACACACATACGACTAC	CAGACCCATCCAGTAACAAGAC
*FOXJ1*	TGAAGCCACCCTACTCCTAT	GTTGTCCGTGATCCACTTGTA
*MUC5ac*	CGATGTGTAGCCAGGATTGT	GTGGCGTGGTAGATGTAGATAG

## Data Availability

The datasets used and/or analyzed during the current study are available from the corresponding author on request.
